# Synthesis, molecular docking, and pharmacological evaluation of 5-(4-(2-(5-ethyl pyridine-2-yl) ethoxy) benzyl)-3-(phenylsulfonyl) thiazolidine-2, 4-dione against HFD-induced diabesity via interaction with the CB1 receptor

**DOI:** 10.22038/IJBMS.2022.65649.14443

**Published:** 2022-08

**Authors:** Farah Deeba, Mohammad Shahar Yar, Mohammad Rafi Haidar, Arun K. Sharma, Manju Sharma

**Affiliations:** 1 Department of Pharmacology, School of Pharmaceutical Education and Research, (SPER) Jamia Hamdard, Delhi-110062, India; 2 Department of Pharmaceutical Chemistry, School of Pharmaceutical Education and Research, (SPER) Jamia Hamdard, Delhi-110062, India; 3 Lord Buddha Koshi Pharmacy College, Saharsa, Bihar, India; 4 Department of Pharmacology, Amity University Haryana, Gurugram-122413, India

**Keywords:** Cannabinoid receptor, Diabesity, Diabetes mellitus, Endocannabinoid, Molecular docking, Obesity

## Abstract

**Objective(s):**

CB1 antagonism arbitrates a dormant shape to the endocannabinoid system that alleviates diverse pathological incidents of diabesity. The present study pursued the synthesis and evaluation of thiazolidine derivative (BAC) having pleiotropic action on CB1R, with or without AM251 (selective antagonist of the CB1 receptor) against high-fat diet (HFD) induced diabesity in C57BL/6 mice.

**Materials and Methods:**

A molecular docking study for CB1 antagonistic potential was conducted by Maestro 11.4 program (Schrodinger Inc., USA), and the thiazolidine derivative BAC was synthesized. The assessment of varied parameters including anthropometric, neurobehavioral, hyperglycemia, dyslipidemia, oxidative stress, and inflammatory cytokines was evaluated in HFD-fed animals as compared with individual and combined treatments of BAC and AM251.

**Results:**

Incomparable to AM251, the treatment of BAC was reported for a significant reduction in food intake and obesity, diabetic biomarkers, lipid profile, oxidative stress, and proinflammatory cytokine release. Moreover, the BAC treatment showed no significant alteration in neurobehavioral activity, including anxiety and depression.

**Conclusion:**

The preliminary in silico study suggests that BAC has a close interaction with CB1 antagonism but has no sign of neurobehavioral alteration. Simultaneously, this compound showed significant ability to ameliorate diversity by the underlying mechanisms of minimizing oxidative stress, regularizing the lipid profile, and reducing pro-inflammatory cytokines.

## Introduction

Diabesity is a rising outbreak across the globe that represents the coexistence of diabetes and obesity. Contemporary data imparts the towering prevalence of metabolic syndrome where about 463 million adults are suffering from diabetes mellitus and is further estimated to escalate up to 592 million by 2035 and 700 million by 2045 (1, 2). Additionally, the incidence of obesity has also been increasing gradually among younger (40.0%), middle-aged (44.8%), and older adults (42.8%) in the past decades (3). Although a wide range of therapeutic agents is available in clinical practice for curing metabolic disorders, conversely, the occurrence of these insidious diseases is still growing extensively. Thiazolidine-2,4-dione (TZD) is an elite platform of magnificent heterocyclic moiety (commonly known as glitazones group) for drug discovery and could upsurge antidiabetic potential. Reports also revealed TZD-associated improved endothelial function, upsurge of adiponectin levels, muscular uptake of insulin-dependent glucose, and diminishing of insulin resistance and hepatic gluconeogenesis (4). TZD could bind with nuclear transcription regulators (peroxisome proliferator-activated receptor-gamma, PPAR-gamma) and influence gene expression. This class has huge margins of safety and significant potency that urge a need for further investigation into this class (5). Numerous reports reveal the molecular nexus of the cannabinoid-1 receptor (CB1R) that could produce significant weight loss and improved glucose tolerance (3). The Endocannabinoid system (ECS) participates in the development of obesity and diabetes mellitus through varying consistent factors including glucose metabolism, lipid metabolism, and insulin sensitivity (6). Our previous report endorses the involvement of the ECS system in pathological events of diabesity (3). Rimonabant was the first approved inverse agonist of CB1R that had significant therapeutic effects on obesity and glucose tolerance by blocking the receptor sites present in the brain, liver, pancreas, and muscles (7). However, rodent models of diabetics showed that Rimonabant and Ibipinabant had beneficiary effects on pancreatic islets for regulating blood glucose levels and insulin secretion (3, 7, 8). While this magic drug was withdrawn from the market after revealing its detrimental influence on the neurobehavioral system, including severe depression and suicidal tendencies (3). Conversely, its withdrawal opens a new doorway for investigating the potent substitute to preside over the EC system. Over the last decade, diverse CB1 receptor antagonists (selective/nonselective) were investigated for probing their pharmacological potential (3). CB1 antagonists and inverse agonists have been reported to increase islet functions during overactive ECS or at increased glucose levels (8, 9). Furthermore, the activation of hepatic CB1R was observed in insulin resistance, impaired metabolic function, and obesity, either by the upsurge of energy intake, glucose impairment, lipid metabolism, or increased reactive oxygen species and cytokines release (3, 7–9). Moreover, impeding of peripheral CB1R recovers insulin sensitivity and glucose metabolism and restrains body weight in animal models (7–10). Hence, investigation of EC receptors targeting CB1R may postulate a potent strategy for the treatment of diabesity. Although existing scientific evidence endorses numerous health benefits of glitazones and diminishes premature death. But the molecular interaction of glitazones with CB1R and their associated therapeutic effects is still unexplored and provides dynamic grounds for the present investigation. The current study was intended to explore the effect of a newly synthesized derivative of thiazolidine with its pleiotropic action on CB1R against high-fat diet-induced diabetes and obesity in C57BL/6 male mice. A selective antagonist of the CB1 receptor (AM251) was used to observe the comparative antagonistic potential of BAC. The compound was synthesized and studied for its therapeutic potency centralizing its neurobehavioral effect, lipid profile, oxidative stress, and inflammatory cytokines.

## Materials and Methods

***Compound synthesis***

**Figure 1:**
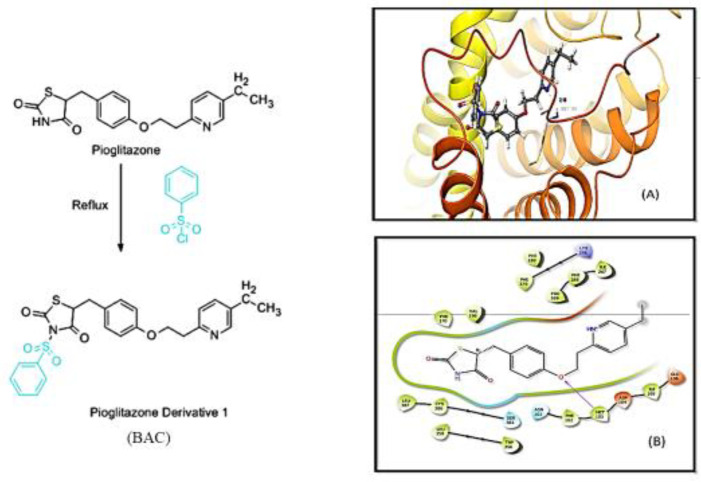
Represents the scheme of synthesis of thiazolidinedione derivative “BAC” (5-(4-(2-(5-ethyl pyridine-2-yl) ethoxy) benzyl)-3-(phenylsulfonyl) thiazolidine-2,4-dione). 2D structure of molecular docking of BAC (A) and 3D structure of BAC (B)

Thiazolidine was reacted with benzene sulfonyl chloride to form its derivative 5-(4-(2-(5-ethyl pyridine-2-yl) ethoxy) benzyl)-3- (phenylsulfonyl) thiazolidine-2,4-dione or test compound BAC (scheme has given in Figure 1). In brief, the alcoholic solution of pioglitazone (3.56 g in 100 ml) and benzene sulfonyl chloride (1.3 ml) was mixed drop by drop, and the reaction mixture was refluxed for 3-4 hr. The reaction was monitored using thin-layer chromatography. On completion, the reaction mixture was allowed to cool down at room temperature and then poured into an ice-water mixture with continuous stirring. The white precipitate obtained was filtered, washed with cold water, and then dried and crystallized from ethanol. 

***In-silico*** **analysis**

*in silico *analysis of synthesized compounds (BAC) for determining CB1 antagonistic potential was conducted using Maestro 11.4 program (Schrodinger Inc., USA) to establish various interactions between the test compound and the target protein. A molecular docking study was performed on 3D X-ray crystal structures of human Cannabinoid receptor 1 (CB1) bearing an internal ligand (PDB ID: 5TGZ) retrieved from the protein data bank. Structure-based modeling was started using protein preparation wizards for virtual screening enrichments. All possible and pre-processed ligand residues, different chains, and water molecules were identified and removed. The structure was further optimized for hydrogen bonding followed by energy minimization. The interaction energy between ligands and receptors was achieved through grid mapping where probe molecules were placed around the target protein. The coordinates were observed by grid maps of interaction energies between assigned ligands and the surrounding protein atoms. Furthermore, it also provides an opposite surface topology for ligand atoms to interact with the binding site and helps GLIDE docking calculations to determine the total interaction energy. Eventually, the most suitable binding site for ligand docking was located by the receptor grid (20 Å × 20 Å × 20 Å) that was generated using the Receptor Grid Generation tool embedded in Glide software. 

***Animal experimental protocol***

C57BL/6 mice with an average weight of 20 ± 5 g were used in this study, which was approved by the animal house facility of Jamia Hamdard, Delhi (IAEC approval No: 10/2/2020/1685). All animals comprising the present study were housed under uniform laboratory conditions, temperature (22 + 2 ºC), and ambient humidity (60% ± 10%) with access to food and water ad libitum in the normal light-dark cycle (light for 700 hr). Diabesity was developed by using a high-fat diet (HFD) having constituents including 45.5% standard chow, 22.7% lard, 22.7% vegetable shortening, and 9% sucrose which can be stored in the refrigerator (temperature 7 ± 2 °C) for 5 days. Conversely, the standard diet for the control group contained 100% chow (11). HFD and standard diet provided ⁓6.5 Kcal g−1 and 3.25 Kcal g−1, respectively.

A total of 48 animals were equally divided into 6 subgroups (8 animals in each group) including the normal control group, HFD group, BAC *per se* group, AM251 (selective antagonist of the CB1 receptor) treatment, BAC treatment, and combined (AM251 and BAC) treatment group.

1. Normal control groups received no treatment with free access to standard food and water during the complete protocol of investigation (24 weeks).

2. The HFD group was considered a disease control group in which animals received free access to self-prepared HFD and water *ad libitum* for 24 weeks.

**3.** *Per se* group received only BAC (20 mg/kg*/p.o*) for 24 weeks in addition to free access to standard food and water.

4. The AM251 treatment group received HFD for 24 weeks, but the treatment of AM251 (1 mg/kg/*s.c* daily for 10 weeks) was simultaneously started after 14 weeks and continued up to 24 weeks.

5. Similar to the AM251 treatment group, the BAC treatment group also received HFD for 24 weeks and simultaneous treatment of BAC (20 mg/kg/*p.o* on a daily basis for 10 weeks) was started after 14 weeks and continued up to 24 weeks.

6. Moreover, the combined treatment of AM251 (1 mg/kg/*s.c* on a daily basis for 10 weeks) and BAC (20 mg/kg/*p.o* on a daily basis for 10 weeks) was given to HFD-fed animals after 14 weeks and continued up to 24 weeks.

***Physiological, biochemical, and histological analysis***

Anthropometrical parameters including body weight and food intake (reflect energy intake) were monitored weekly. Abdominal circumference and body mass index (BMI) of animals of respective groups were calculated to measure adiposity. 

The toxicological impact of test drugs (BAC and AM251) on neurobehavioral permanency was studied based on analysis through the elevated plus-maze test (EPMT), forced swim test (FST), and sucrose preference test (SPT). Additionally, structural changes were observed in histological images of brain tissue. Effect on FST (to assess depression) and EPMT (to assess anxiety-related behavior) were assessed for measuring the behaviors of depression and anxiety, respectively in the mouse model, where FST was performed in a cylindrical plastic drum with a diameter of 45 cm and depth of 40 cm filled with tap water of 30±2 **°C** temperature. EPMT (for 5 min duration) was carried out with the help of an elevated plus-maze with standard dimensions for mice. Video recording was done for scoring different behaviors, including entries and percentage time spent in the open and closed arms of each animal of their respective groups. Diverse behavioral parameters including climbing, swimming, and floating were observed for assessing FST. After each trial, mice were dried with a cotton towel and were returned to their home cages (12).

Moreover, behavioral manifestations including anhedonia which indicate the non-somatic symptoms of depression were reported by analyzing SPT. The mice were water-deprived for 18 hr and then each mouse was exposed to two bottles in each cage (one of pure water and the other of sucrose solution (1%)). Water consumption of the animals was measured and percentage sucrose preference was calculated (13):

[Sucrose solution intake (g) / total intake (g)] **×** 100

Where total intake = sucrose solution intake and tap water intake 

All mice were given adaptive training during the 4 days for SPT (13).

Biochemical parameters include oral glucose tolerance test (OGTT), blood glucose levels (using commercially available glucometers (AccuChek Active Blood Glucose Meter Kit)), serum insulin (using a commercial ELISA kit (Mercodia, Uppsala, Sweden)), HbA1c (using a commercial ELISA kit (Crystal Chem Inc Downers, USA)) and HOMA-IR (homeostasis model assessment of insulin resistance) were investigated for endorsing the development and presence of diabetes. The OGTT was performed after administration of a dose of glucose of 2 g/kg (14). Animals were fasted for an overnight period and blood samples were withdrawn from the tail vein puncture at 0, 30, 60, 90, and 120 min.  For serum sample preparation, blood was collected from animals of respective groups by puncturing the retro-orbital sinus of mice using a sterile hematocrit capillary tube, and serum was isolated by centrifugation for 15 min at 2000 g. The HOMA-IR index was calculated to assess insulin resistance by using the given formula (15):

Fasting serum glucose × fasting serum insulin/22.5 

Diabesity-associated dyslipidemia and atherosclerosis were calculated by estimating total cholesterol (TC), low-density lipoprotein (LDL), very-low-density lipoprotein (VLDL), and high-density lipoprotein (HDL), triglycerides (TG), and coronary risk index (CRI). All lipid parameters were probed using a commercial ELISA kit (ERBA Diagnostics, Inc) (16). In addition, the structural arrangement of hepatic cells was examined by histological evaluation. Furthermore, antioxidant and anti-inflammatory parameters were analyzed by assessing TBARS, SOD, catalase, and IL-6, TNF-α, as mentioned in our previous reports (1, 17).

All isolated tissues (brain, liver, and pancreas) for histology were stored in a 5% formalin solution and processed for cutting, fixing, and staining. Hematoxylin-eosin (H&E) staining was applied to highlight the nucleus and cytoplasm sections, and histological images were reviewed under the microscope.

***Statistical analysis***

All data were presented as mean ± SD. Statistical differences were calculated by using one-way ANOVA followed by Tukey’s multiple comparison tests using Sigma Plot version 11.0, from Systat Software, Inc., San Jose California, USA. A *P*-value of <0.001 was reflected as statistically significant.

## Results

***Compound synthesis and in silico interaction***

The synthesis of test compound BAC or 5-(4-(2-(5-ethyl pyridine-2-yl) ethoxy) benzyl)-3- (phenylsulfonyl) thiazolidine-2,4-dione was carried out by the reaction of pioglitazone with benzene sulfonyl chloride as shown in the scheme (Figure 1). The melting point of recrystallized transparent crystals of the title compound (BAC) was determined by the open capillary tube method and was found to be 204–206 **°**C. 1H-NMR of the synthesized compound was performed to confirm its chemical structure.

The physical state and yield were measured as 62% and melting point 204–206 °C, respectively. Electronic environment of chemical equivalent and non-equivalent protons were studied by 1H NMR (300 MHz, CDCl3, δ ppm): 1.21-1.26 (3H, t, CH3), 2.59-2.67 (2H, m, CH2), 3.20-3.25 (2H, m, CH2), 3.39-3.45 (2H, m, CH2), 4.29-4.33 (1H, t, CH; thiazolidinedione ring), 4.45-4.50 (2H, m, CH2), 6.83-6.86 (2H, d, Ar.H), 7.10-7.13 (1H, m, Ar.H), 7.18-7.20 (2H, m, Ar.H), 7.26 (1H, s, Ar.H), 7.44-7.48 (5H, m, Ar.H), 8.39 (1H, s, Ar.H; pyridine ring). 

A noncovalent three-dimensional (3D) interaction between the CB1 receptor and the BAC (thiazolidinedione derivative) test compound showed good docking scores comparable to other derivatives including AM251 (Table 1). Molecular docking of test compounds (BAC) and AM251 also showed similar binding pockets and exhibited similar hydrogen bond interactions with SER 383.  

***Effect of test drugs on neurobehavioral parameters***

**Figure 2:**
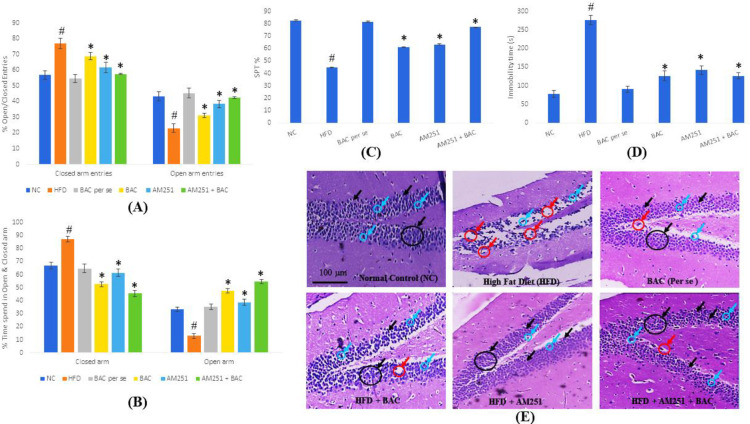
Describes the effect of BAC and AM251 on neurobehavioral parameters including the percentage of open and closed entries (A), percentage of time spent in the open and closed arm (B), percentage of sucrose preferences test (C), and time duration of immobility during FST (D). All values were expressed as mean ± SD whereas, ‘#’ represents *P*<0.001 vs Normal Control; ‘*’ represents *P*<0.001 vs HFD-fed group. (E) Reveals the histology of small pyramidal cells of the hippocampus (CA1) region with vesicular nuclei in diverse groups of treatments using an inverted microscope (Cosmo Laboratory Equipment). The black arrow shows granule cells of the dentate gyrus. The red arrow shows vacuolization and cell death, and the blue arrow shows glial cells

Assessment of neurobehavioral parameters is a widespread valuation of both neurological integrity and behavioral function. Neurobehavior analysis in mice has been used to assess psychological processes. These assessments revealed the toxicological effects of BAC and AM251 treatments, if any, as they are considered CB1 receptor antagonists. Anxiolytic effects of the given drug in diabetic and obese mice were examined by EPMT, FST, and SPT. For EPMT, the % entries in open and closed arms with 5-minute time slots were calculated. The % entries in the closed arm were higher (or lower in the open arm) in HFD-fed animals as compared with normal control animals. Whereas the individual and combined treatments of BAC and AM251 showed a significant decline in % entries of the closed arm (substantial intensification in % entries of the open arm) in HFD-fed animals as compared with the animals who only received HFD (Figure 2). Moreover, the immobile state during FST indicates the depression and helplessness of animals. A long-time duration of immobility was noticed in HFD-fed animals as compared with normal control. The duration of this immobility state was significantly less in the treatment groups (BAC and AM251 individually and in combination) as compared with HFD-fed animals (Figure 2). A prominent diminution in pleasure activities significantly reveals the symptoms of mood disorders and psychosis (anhedonia) that can be associated with other psychiatric and neurodegenerative disorders. The % value of SPT was significantly reduced in HFD-fed animals as compared with normal control. The individual treatment of BAC and AM251 moderately increased the % SPT value. Whereas the combined therapy significantly surges the % sucrose preference value (Figure 2). 

***Effect of test drugs on physiological and biochemical parameters***

The consequences of physiological parameters including food intake, body weight, BMI, and abdominal circumference were monitored throughout the experimental protocol duration. Food intake (food consumption) on a daily basis was significantly increased in HFD-treated animals as compared with normal control. Whereas the food intake of the normal control group was comparable to the BAC *per se* group. Animals treated with BAC and AM251 showed significantly less food consumption as compared with HFD-fed animals. A similar observation was found in the group of combined treatments of BAC and AM251 against HFD-fed animals (Table 2).

The cumulative increase in body weight gain and abdominal circumference was observed in mice having HFD for 24 weeks as compared with normal control mice. Conversely, there was no significant change in body weight gain and abdominal circumference for normal control and HFD-fed animals. Individual and combined treatment of BAC and AM251 significantly reduced mean body weight and abdominal circumference in the respective groups. BMI was further calculated to measure obesity in HFD-fed animals and the effect of respective treatments. The negotiable alteration was observed in BMI of normal control and BAC *per se* groups, whereas a substantial increase in BMI was observed in HFD-fed animals as compared with normal control. The treatment of BAC and AM251 either in individual or in combined therapy significantly reduced obesity and a condensed BMI was observed as compared with HFD-fed animals (Table 2). 

**Figure 3:**
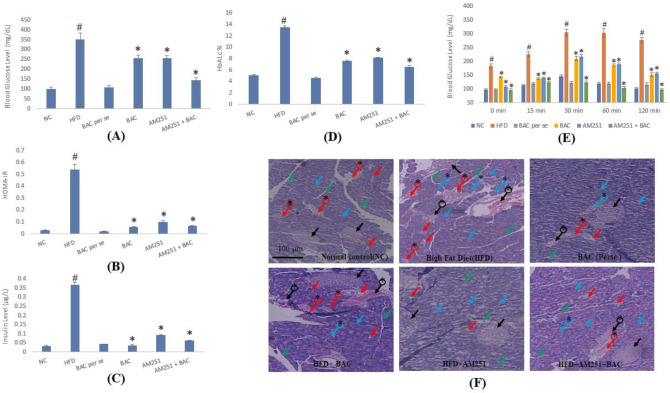
Figure depicts the effect of BAC and AM251 on blood glucose (A), HOMA-IR (B), serum insulin (C), percentage HbA1c (D), and blood glucose level after OGTT at 0, 15, 30, 60, and 120 min (E). All values were expressed as mean ± SD whereas, ‘#’ represents *P*<0.001 vs Normal Control; ‘*’ represents *P*<0.001 vs HFD-fed group. (F) Reveals the histological architecture of pancreatic tissue in diverse groups of treatments. The black arrow shows the circular shape of the islets of Langerhans. Blue arrow shows packed in acinar cells. The red arrow shows pancreatic intact intralobular septa present in exocrine components. The green arrow shows the intact interlobular septa. Black arrow with * shows damage to or entire loss of islets of Langerhans. The Blue arrow with * shows swelled acinar cells with small vacuoles, and the red arrow with * shows flattened interlobular duct

HFD: high-fat diet; OGTT: oral glucose tolerance test

The glucose tolerance test was performed after administration of a 2g/kg dose of glucose and blood glucose level was measured after 0, 15, 30, 60, and 120 min. The restriction in the rising of blood glucose levels reflects the tolerance ability of the animal or glucose homeostasis. At 0 min, the significant rise in blood glucose was slightly less, except in HFD-fed animals. This moderate increase was further reversed by treatment therapy with BAC and AM521 (either alone or in combination). In addition, the increase in glucose levels was insignificant in the normal control verses drug *per se* group. A similar pattern of rising in blood glucose levels was measured in all respective groups at different time slots including 15th, 30th, 60th, and 120th min with a high surge of blood glucose levels in HFD-fed animals as compared with normal control and drug *per se* groups. BAC and AM521 caused a significant restriction in increasing blood glucose levels in HFD-fed animals either alone or in combination. However, the potential for combined therapy was significantly more successful (Figure 3). 

Moreover, long-term intake of the HFD diet caused alteration in glucose homeostasis and insulin sensitivity in animals. The consequence of the present investigation endorses the outcomes of previous studies where a significant rise in blood glucose levels was measured in HFD-fed mice as compared with normal control groups. BAC and AM251 treatment, either alone or in combination produced a substantial reversal of increased glucose levels in HFD-fed animals. There was no difference observed in glucose levels of the normal control group and BAC per group.  The same effect was observed in serum insulin, HOMA-IR, and HbA1c levels in the 10-week treatment of BAC and AM251 groups (both alone or in combination) against HFD-fed animals as compared with normal control and drug *per se* group (Figure 3). 

**Figure 4:**
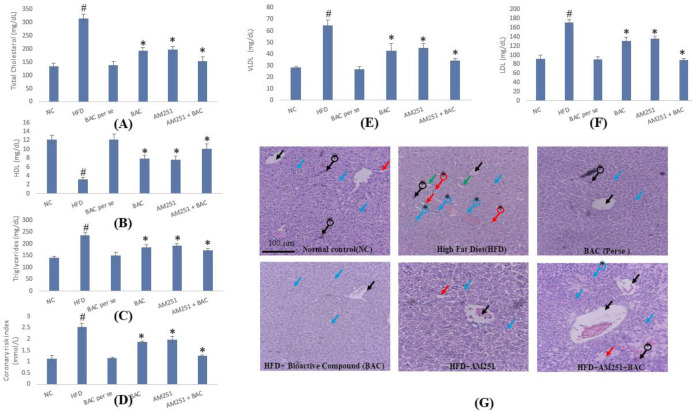
Effect of BAC and AM251 on total cholesterol (A), HDL (B), Triglycerides (C), Coronary risk index (D), LDL (E), and VLDL (F). All values were expressed as mean ± SD whereas, ‘#’ represents *P*<0.001 vs Normal Control; ‘*’ represents P<0.001 vs HFD-fed group. (G) Reveals the histological architecture of hepatic tissue in diverse groups of treatments. The black arrow shows the central vein. Blue arrow shows the hepatic cords. The red arrow shows swelling of hepatocytes. The green arrow shows the Kupffer cell activation. A black arrow with * shows karyomegaly. Blue arrow with * shows portal infiltration with inflammatory cells. A red arrow with * shows hyperplasia and hyperactivation of the epithelial lining bile duct

HDL: high-Density Lipoprotein; LDL: low-density lipoprotein; HFD: high-fat diet

Dyslipidemia is another root cause of various fatal complications that would be pointedly associated with diabetes and obesity. Thus, the present study involved the analysis of lipid profiles where the consequences revealed a significant rise in total cholesterol, low-density lipoprotein, very-low-density lipoprotein, and triglyceride in HFD-fed animals as compared with normal control and drug *per se* groups. The treatment of BAC and AM251 either in individual therapy or in combination significantly slows down the increase of these lipid parameters. Although combined therapy has a potent influence on lipid parameters as compared with individual treatment. Moreover, a significant reduction in high-density lipoprotein was observed in HFD-fed animals as compared with the normal control group. The treatment with BAC and AM251 moderately increased HDL levels (Figure 4). Additionally, combined therapy treatments significantly increased the level of HDL as compared with individual treatment. Overall, the outcomes of lipid profile parameters indicate that the treatment of BAC and AM251 significantly overcomes dyslipidemia by diminishing bad cholesterol and upsurging good cholesterol levels in HFD-fed animals. Also, the associated vascular damage of dyslipidemia was assessed by calculating the CRI. The level of CRI was comparatively higher in HFD-fed animals than in normal control and drug *per se* groups. However, the coronary risk index was improved by the BAC, AM251, and BAC+AM251 groups, respectively, in contrast to the HFD group (Figure 4).

***Effect of test drugs on oxidative stress and inflammatory cytokines***

**Figure 5:**
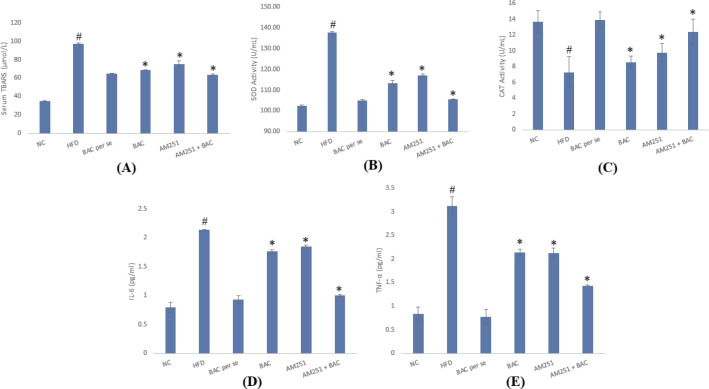
Portrays the effect of BAC and AM251 on serum TBARS (A), SOD activity (B), CAT activity (C), IL-6 (D), and TNF-α (E). All values were expressed as mean ± SD, whereas ‘#’ represents *P*<0.001 vs Normal Control; ‘*’ represents *P*<0.001 vs HFD-fed group

Oxidative stress plays a crucial role in the pathological signaling of chronic diseases, including neurobehavioral, vascular, and metabolic disorders (18). The evaluation of oxidative stress was performed by determining the levels of TBARS content, superoxide dismutase, and catalase activity. The levels of serum TBARS and SOD activity were found to be significantly higher in HFD-fed animals as compared with the normal control group. The BAC *per se* group showed insignificant changes in TBARS and SOD as compared with the normal control group. Conversely, the treatment of BAC and AM251 significantly reduced the level of TBARS and SOD activity by individual and combined treatment (Figure 5). Inversely, the level of CAT activity was significantly reduced in HFD-fed groups in comparison with the control group. The individual and combined treatment of BAC and AM251 upsurge the CAT activity in HFD-fed animals (Figure 5).

Proinflammatory cytokines, including IL-6 and TNF-α are released by adipocytes and their increased levels are associated with the dissemination of fatty cells (19). These proinflammatory cytokines endorse the production of acute-phase proteins and inflammatory events. In the present investigation, the level of these proinflammatory cytokines was significantly raised in HFD-fed animals as compared with normal control. BAC *per se* showed no significant changes in comparison with the control group. Whereas the treatment groups (BAC, AM251, and combined treatment of BAC+AM251) had significantly diminished release of cytokines (Figure 5). 

***Effect of test drugs on structural arrangements of brain, liver, and pancreas***

Histological sections of different vital organs of the respective groups showed diverse structural variations. The exposed area of brain tissue represents the histology of small pyramidal cells of the hippocampus (CA1) region with vesicular nuclei. Histological images of the normal control group showed the presence of granule cells of the dentate gyrus and enormous numbers of glial cells. The photomicrographs of normal control were comparable with the histology of the BAC *per se* group. The histology of HFD-fed animals represents a large area of vacuolization and cell death. Treatment groups, including BAC, AM251, and combined therapy reversed pathological changes as compared with histology in HFD-fed animal groups. The predominance of granule cells of the dentate gyrus and glial cells was observed in histological sections of combined therapy of BAC and AM251 (Figure 2). The photomicrographs of the histological architecture of pancreatic tissue showed the circular shape of islets of Langerhans, acinar cells, and intact intralobular/interlobular septa in the normal control and drug *per se *group. On the other hand, pancreatic tissue from the HFD-fed animals showed damage to or entire loss of islets of Langerhans, swelled acinar cells with small vacuoles, and flattened interlobular duct. The histology of treatment of either individual BAC and AM251 or their combined approach significantly diminished the pathological events, and their histological architecture of pancreatic tissue was comparable to normal groups (Figure 3).

In addition, the structural arrangements of hepatic tissue of normal control and drug *per se* revealed the presence of normal central vein and hepatic cords. Whereas numerous pathological alterations were observed in the histology of hepatic tissue of HFD-fed animals, including the abolition of normal central vein, swelling of hepatocytes, Kupffer cell activation, karyomegaly, portal infiltration with inflammatory cell, hyperplasia, and hyperactivation of the epithelial lining of bile duct. The diminishing of these pathological alterations and regeneration of healthy cells were reported in the histology of drug treatment groups (either by individual BAC and AM251 or their combination). This reversal of pathological events showed the protective action of BAC, AM251, and their combined therapy against obesity and diabetes-induced hepatic damage (Figure 4). 

**Table 1:** Molecular Docking Score of Novel test compounds (BAC) as compared with the cannabinoid-1 (CB1) receptor

**Sr. no.**	**Compounds**	**Docking scores (KJ/mol)**
**1**	AM251	-12.66
**2**	Pioglitazone	-10.40
**3**	Pioglitazone Der. (BAC)	-10.19

Sr.no: Serial Number

**Table 2:** Daily monitored physiological parameters including food intake, bodyweight, abdominal circumference, and body mass index (BMI) of mice

**Sr. no.**	**Experimental groups**	**Daily food intake (g)**	**Body weight (g)**	**Abdominal circumference ** **(mm)**	**BMI (g/cm** ^2^ **)**
**1**	**Normal control**	63.30 ±8.12	23.72 ±0.36	63.22 ±6.87	3.3±0.19
**2**	**HFD**	86.74 ±11.3#	45.24 ±1.55#	88.82 ±7.34#	5.5±0.31#
**3**	**BAC ** ***per se***	64.38 ±10.82	24.15 ±1.82	63.78 ±8.42	3.2±0.27
**4**	**BAC**	67.88 ±8.82*	35.88 ±1.22*	69.58 ±7.07*	4.7±0.17*
**5**	**AM251**	68.96 ±7.12*	35.24 ±1.78*	72.48 ±5.16*	4.3±0.11*
**6**	**AM251 + BAC**	63.08 ±7.63*	27.11 ±1.18*	61.58. ±8.32*	3.4±0.31*

All values were expressed as mean ± SD whereas, ‘#’ represents *P*<0.001 vs Normal Control; ‘*’ represents *P*<0.001 vs HFD-fed group

HFD: high-fat diet

TBARS: thiobarbituric acid reactive substances

## Discussion

Diabetes and obesity are the most interconnected and protruding metabolic disorders which include alteration in glucose metabolism followed by insulin resistance and insulin deficiency that could trigger numerous fatal complications (1). Diabesity could significantly provoke nephropathy, cardiomyopathy, neuropathy, dyslipidemia, inflammation, hypertension, and visceral adiposity (20). Although numerous classes of medicine are available in the market for treating diabetes mellitus, no such pharmacological intervention has been reported to date for treating obesity and its associated complications. Over a decade, endocannabinoids have been studied for their potent ability to suppress appetite and regulate obesity as well as diabetes mellitus (21). In this series, the potent inverse agonist (Rimonabant or SR141716A) of CB1R was discovered and approved for the treatment of obesity and improved glucose tolerance (2, 7, 22). Afterward, the drug was withdrawn from the market due to its serious side effects on neurobehavioral functions (2, 7, 22). Although this promising anti-obesity drug was removed from the market, it also opened a new window of research where several investigations have been carried out for finding some other novel CB1R antagonists. Rimonabant was withdrawn because of its neuropsychiatric events as it could cross the blood-brain barrier. AM251 has been reported for CB1 antagonism and reduced appetite (23). These properties of AM251 make it equally potent as Rimonabant, with additional auspicious consequences of no deleterious impact on neurobehavioral action (23, 24). Moreover, a recent report revealed that pyrazole-based drugs may have CB1 antagonistic potential (25). The combined therapy of antidiabetic drugs and CB1 antagonists was also reported to lessen diabesity (2, 8, 26). In the present investigation, we used a thiazolidine derivative (BAC) that was synthesized by the reaction of pioglitazone with a benzene sulfonyl chloride. The structure of BAC was pre-evaluated for its noncovalent three-dimensional (3D) interaction with CB1R. This *in silico* report confirms the maximum interaction between probe and receptor endorsed by docking score as compared with other derivatives, including AM251. The interaction of the ligand with CB1R increased the probability of the neurotoxic effect and thus, the neuro-toxicological impact of BAC needed to be explored. The consequences showed that BAC treatment had no detrimental effect on neurobehavioral activity, including anxiety, depression, and psychosis (anhedonia). Conversely, the report suggests that BAC has similar potency as AM251 for anxiolytic and anti-depression activity. Further, the synthetic compound was evaluated for its therapeutic potential with or without AM251 (a CB1R antagonist). As discussed, the experimental protocol was developed in C57BL/6 mice for developing diabesity. The anthropometric parameters including bodyweight, abdominal circumference, and BMI of investigating mice revealed the presence of diabesity in HFD-fed animals (27). The consequence supports the presence of diabetes and obesity as suggested by previous reports (27). The measurement of reduced blood glucose, insulin levels, OGTT, improved HbA1c, and improved HOMA-IR in the individual and combined treatment of BAC and AM251 were reported with prompt and faster reduction of blood glucose and insulin levels. These outcomes revealed the anti-diabetic potential of BAC and AM251. Diabetes mellitus and obesity are the root causes of developing dyslipidemia, oxidative stress, and inflammatory effects (2, 28). The present outcomes reported similar consequences where disruption of the level of lipid biomarkers was observed. Previous reports have evidenced that increase in levels of TC, LDL, VLDL, and TG and reduced HDL favors the incidence of dyslipidemia (29). The occurrence of dyslipidemia, diabetes mellitus, and obesity eventually leads to interference in vital organs by aggregate, prevalence of reactive oxygen species, and inflammatory cytokine release (1, 11, 16). The improvement in coronary risk factors by BAC and combined treatment with AM251 showed the protective potential of BAC against atherosclerotic cardiovascular disease. The individual and combined therapy of BAC and AM251 revealed the ameliorating potential against HFD-induced diabesity and associated dyslipidemia, oxidative stress, and inflammatory cytokine release. Furthermore, the histological architecture of vital organs validates the alteration measured by biochemical estimation (30). The structural arrangements of brain tissue, including large areas of vacuolization and cell death represent the pathological impact of diabesity on HFD-fed animals (30). Whereas no alteration in the histology of animals of the drug *per se* group indicated no toxic impact on neuronal tissue. The lowering of these pathological changes in pyramidal cells of the hippocampus (CA1) region with vesicular nuclei in the BAC and AM251-treated group confirmed the protective action. Correspondingly, the entire loss of islets of Langerhans swelled acinar cells with small vacuoles, and flattened interlobular duct in pancreatic tissue of HFD-fed animals endorse the presence of diabetes mellitus (30, 31). However, the presence of circular shape of islets of Langerhans, acinar cells, and intact intralobular/interlobular septa in normal control and the drug-treated group showed their anti-diabetic potential. Additionally, the destruction of normal central vein, swelling of hepatocytes, karyomegaly, portal infiltration with inflammatory cells, and hyperactivation of the epithelial lining of bile duct in hepatic tissue of HFD-fed animals showed hepatic damage (11). Conversely, the reversal of these pathological events and the presence of normal central vein and hepatic cords in the drug-treated group strengthen the hepatoprotective potential of BAC and AM251. 

## Conclusion

The present report revealed the new thiazolidine derivative that may have pleiotropic CB1R antagonistic activity. The compound has significant potential against HFD-induced diabesity with no neurobehavioral impairment. Furthermore, the combined BAC and AM251 treatment was comparatively more potent than the individual approach. This study does not confirm the protective action of BAC through CB1R antagonism, but, *in silico* results favor the fact. The possible underlying mechanism of this protective action of BAC may be the dissuasion of oxidative stress, and inflammatory cytokine release and might be through blocking of CB1R. 

## Authors’ Contributions

FD, MS, and AKS Contributed to the conception and design of the work, interpretation of results, and manuscript content. MSY and MRH Conducted the molecular docking study and data analysis. The final drafting of the work was written by FD, MS, and AKS. All authors read and approved the final manuscript.

## Compliance with Ethical Standars

Ethics approval: Experiments were approved by CPCSEA, New Delhi, India (IAEC approval No: 10/2/2020/1685) and the institutional animal ethical committee.

## Conflicts of Interest

We confirm that there are no conflicts of interest declared by any authors.
